# A novel poxvirus isolated from an Egyptian fruit bat in Israel

**DOI:** 10.1002/vms3.233

**Published:** 2020-02-25

**Authors:** Dan David, Irit Davidson, Asaf Berkowitz, Sharon Karniely, Nir Edery, Velizar Bumbarov, Orly Laskar, Ron Elazari‐Volcani

**Affiliations:** ^1^ Kimron Veterinary Institute Bet Dagan Israel; ^2^ Department of Infectious Diseases IIBR Ness Ziona Israel; ^3^ School of Zoology Tel Aviv University Tel Aviv Israel

**Keywords:** Egyptian fruit bat, Israel, IsrRAPXV, Novel pox virus, *Rousettus aegyptiacus*, Skin lesions

## Abstract

An Egyptian fruit bat (*Rousettus aegyptiacus*) from the Zoological Gardens, at Tel Aviv, Israel, showed pox‐like clinical signs including vesicular and nodular skin lesions on the wings. Cell culture isolation, histopathology, electron microscopy and molecular analysis, revealed the presence of a novel bat poxvirus. Future research is needed to determine whether this virus can affect human health.

## Graphical abstract

An Egyptian fruit bat (Rousettus aegyptiacus) from the Zoological Gardens, at Tel Aviv, Israel, showed pox‐like clinical symptoms including vesicular and nodular skin lesions on the wings. Cell culture isolation, histopathology, electron microscopy and molecular analysis, revealed the presence of a novel bat poxvirus. Future research is needed to determine whether this virus can affect human health.

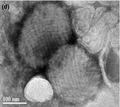

## 1 INTRODUCTION

Bats are a highly diverse order of mammals that are recognized as a reservoir for emerging viruses such as Severe Acute Respiratory Syndrome (SARS), Middle East Respiratory Syndrome (MERS), Nipa, Hendra, Lyssaviruses and filoviruses (Calisher, Childs, Field, Holmes, & Schountz, 2006; Changula et al., 2018). Based on their behavioural and physiological traits, bats are subdivided into two suborders, megabats and microbats. In Israel, *Chiroptera* is the largest mammalian order and contains 33 bat species. The Egyptian Rousette bat (*Rousettus aegyptiacus*), a megabat, a common fruit bat species, found throughout Africa and in the Middle East. The Egyptian fruit bat is the only *megachrioptera* bat in Israel and the most common bat found in urban areas in close proximity to humans, national parks and nature reserves (Levin, 2010).

Poxviruses are double‐stranded DNA viruses with large genomes belonging to the family *Poxviridae*. They are divided into invertebrate‐infecting *Entomopoxvirinae* and chordate‐infecting *Chordopoxvirinae*. Many poxviruses are zoonotic. Poxvirus infected bats has not yet been previously reported in Israel (Levin, 2010) but have been identified in bats elsewhere (Baker et al., 2013 ; Baker & Murcia, 2014; Emerson et al., 2013; McLelland et al., 2013; O’Dea *et al.*, 2016).

The present study reports the first isolation, histopathological and electron microscopic characterization and molecular analysis of a poxvirus isolated from an Egyptian fruit bat in Israel that has provisionally been named *Israel Rousettus aegyptiacus pox virus* (IsrRAPXV).

## 2 CLINICAL PRESENTATION AND SAMPLE PROCESSING

**Figure 1:**
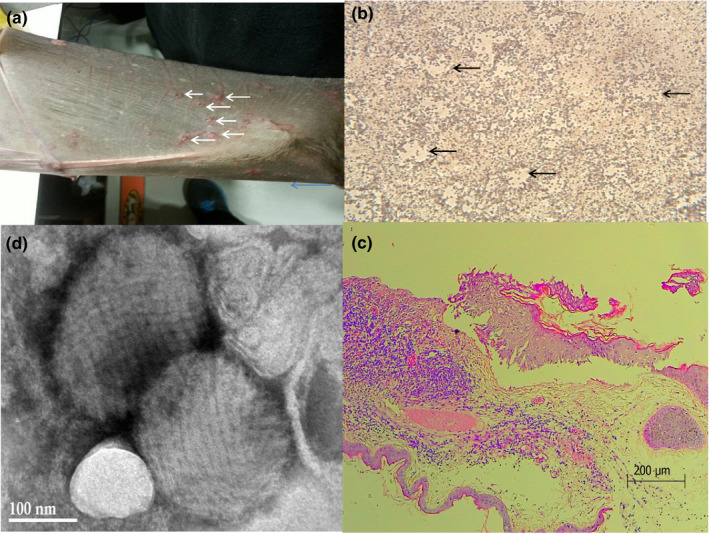
(a) Photograph of the Egyptian fruit bat wing showing multiple poxes‐like lesions (Arrows). (b) Cytopathic effect (Arrows) of IsrRAPXV in Vero cells 6 days post infection (magnification x100). (c) Histologic analysis of lesions from a Egyptian fruit bat *Rosettus Aegiptiacus* infected with novel poxvirus revealing epidermal hyperplasia with ballooning of keratinocytes and the presence of intra‐cytoplasmic inclusion bodies (magnification X 200); (d) Transmission electron micrograph of poxvirus particles with negative staining of cell culture supernatant

On December 2014, an adult female Egyptian fruit bat (*Rousettus aegyptiacus*) from the Zoological Gardens at Tel Aviv University, which had been clinically normal developed pox‐like clinical signs consisting of multiple vesicular and nodular skin lesions on the wing membranes (Figure 1a). The bat was moved from a captive fruit bat colony to an open colony, with unrestricted access to the external environment. A skin biopsy of a nodular lesion and vesicle fluid swabs were collected, before the bat was released into the open colony. The swab specimens were suspended in 2 ml PBS, incubated for 1 hr at room temperature and then clarified by centrifugation at 1,000*g* for 10 min at 4°C. The supernatant was kept at −80°C until DNA extraction could be performed. DNA was extracted (DNeasy, Qiagen, Germany) from the swab sample suspensions and subjected to polymerase chain reaction (PCR) amplifications using pan herpes primers (Ehelers et al., 1999), sheep poxvirus specific primers and lumpy skin disease virus primers (Menasherow et al., 2014). No replication was evident at 40 amplification cycles performed. Cultures for aerobic and anaerobic bacteria were also negative.

Virus isolation was performed using African green monkey kidney epithelial Vero cell cultures (ATCC®, CCL 81™) placed in six‐well plastic plates (Corning, New York, USA). The Vero cell cultures were incubated with samples in 0.5 ml of Phosphate buffered saline (PBS) for 1 hr at 37ºC in an incubator with 5% CO_2_ for viral absorption. Afterwards, 4 ml of maintenance medium (Eagle minimum essential medium, Biological Industries, Bet Haemek, Israel) supplemented with 2% fetal bovine serum (Gibco BRL, Germany), 2% glutamine and 1% antibiotics (Biological Industries, Bet Haemek, Israel) was added and the cell culture returned to the CO_2_ incubator, these cells were inspected daily. Cytopathic effects appeared after 4 days (Figure 1b) at which time supernatant was collected and stored at −80ºC.

Histopathological analysis was performed on the skin biopsy, after it was fixed in 10% formalin. The sample was embedded in paraffin, sectioned and stained with haematoxylin and eosin, according to standard protocols. The skin lesion had a focally extensive marked epidermal hyperplasia with ballooning degeneration, intracytoplasmtic inclusion bodies, ulceration and supportive dermatitis (Figure 1c). These histopathological findings were consistent with poxvirus infection.

The virus was further characterized by transmission electron microscopy (TEM). Media collected from infected cells was centrifuged at 10,000*g* for 20 min at 4ºC and then nuclei and cell debris was discarded. For viral enrichment the supernatant was then overlaid on a 10% sucrose layer and centrifuged at 50,000*g* for 1.5 hr at 4ºC in Sorvall centrifuge with/T1250 rotor (Thermo Scientific, WX ultra 80). Negative staining (Erster et al., 2018) of the enriched virus fraction showed brick‐shaped poxvirus‐like particles with irregular threadlike surface fibres with an average size of 246 nm × 194 nm (Figure 1d).

To test whether that virus was a poxvirus, the DNA that as extracted from tissue culture supernatants, was amplified by PCR using poxvirus consensus primers specific for a 1724 bp fragment of a conserved region of DNA‐dependent DNA polymerase (Tuomi et al., 2014) (Genbank accession no. MK542648). A BLAST search adjusted for a low‐sensitivity mode yielded multiple hits for the DNA polymerase gene of a very diverse collection of poxviruses, all with similar scores. However, the best nucleotide match was 70%‐72% homology, implying that the bat was infected by an unreported and presumably new poxvirus. Further characterization of the new pox virus was performed by PCR using low‐GC content poxvirus primers targeting a 230 bp region of the putative metalloproteinase gene followed by Sanger sequencing (Li, Meyer, Zhao, & Damon, 2010). The top 10 matches to this sequence (GenBank accession no. MK542649), in GenBank were all poxviruses with 76%–78% homology.

**Figure 2:**
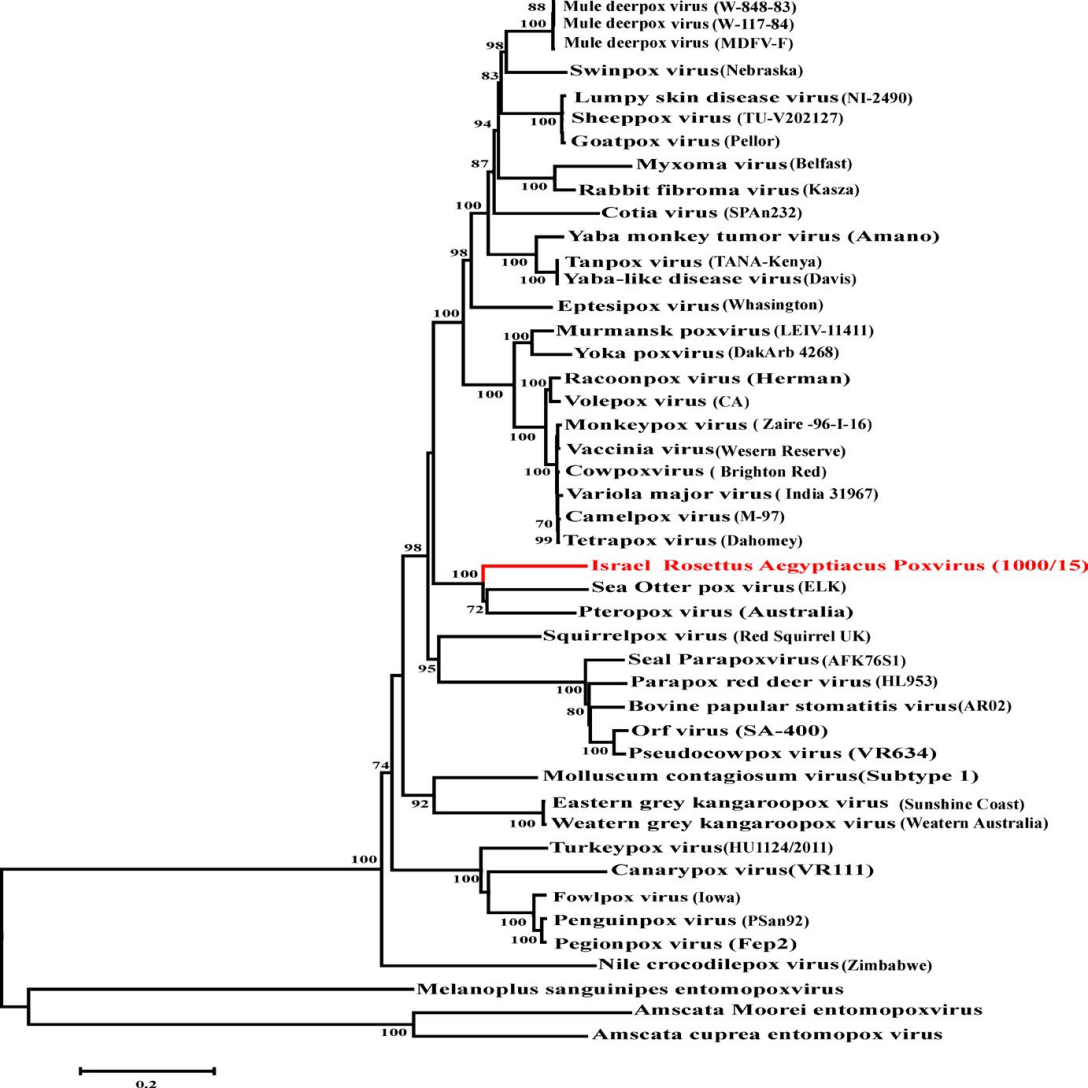
Phylogenetic tree of the poxviridae family. A neighbour‐joining phylogram shows the relationship of the IsrRAPXV (red) to 41 poxviruses based on the concatenated amino acid sequences of seven conserved genes. DNA polymerase, RNA polymerase subunit RPO147, RNA polymerase subunit RPO132, RNA polymerase‐associated RAP94, virion major core protein P4a, early transcription factor VETFL and NTPase (6,659 amino acids characters). Amsacta moorei (red hairy caterpillar) (Moyer), *Amsacta cuprea* (CV6M) and *Melanoplus sanguinipes* (entomopoxviruses) were used as the outgroup sequences. The scale bar indicates amino acid substitutions per site. The strain names are in parentheses

To obtain additional viral sequence for phylogenetic analysis a Next Generation Sequencing on a virus enriched preparation (prepared as described above for TEM analysis) using an Illumina platform (www.illumina.com/technology/sequencing_technology.ilmn). DNA sequences were aligned to the sea otter pox virus (SOPXV accession no. MH427217) (Jacob et al., 2018) and seven open reading frames of our identified virus (037, 069, 073, 081, 096, 099 and 114) (GenBank accession nos. MK542642‐7, and MK542650) were extracted on the basis of sequence similarity. Open reading frames nucleotides (20,435 bp) were translated into amino acid sequences in *silico* (6,659 aa) and then aligned to 41 poxvirus sequences using the Clustal X (v1.83) alignment program. A neighbour‐joining phylogenetic tree was constructed (Figure 2) using the Poisson model of the MEGA program (v6.06). The reliability of the phylogenetic groupings was evaluated using bootstrapping with 1,000 replicates. The phylogenetic tree revealed that pox virus isolated from the Egyptian fruit bat *Rousettus aegyptiacus* in Israel was a novel species of poxvirus that clustered with 100% bootstrap value with poxvirus in the subfamily *Chordopoxvirinae* and that formed a new genera with two recently identified poxviruses, the *Pteropox* virus (PTPV, accession no.NC_030656) and SOPXV (Jacob et al., 2018). The Israeli poxvirus was provisionally designated as IsrRAPXV.

## 3 DISCUSSION

This study describes the isolation and characterization of IsrRAPXV, a new Israeli bat poxvirus from skin lesions of an infected Egyptian Rousette bat *Rousettus aegyptiacus* in Israel. Similar pox‐like skin lesions were described in an Australian little red flying fox (*Pteropus scapulatus*) that was trapped in the Kimberley region of North Western Australia. However, while no viral particles were observed in the homogenized lesion and no cytopathic effect was seen by its cultivation in three cell lines, DNA was isolated from the sample and confirmed the presence of PTPV (O’Dea et al., 2016). Recently, a novel poxvirus was isolated from big brown bats with necrosuppurative osteomyelitis lesions in USA (Emerson et al., 2013). Molecular analysis revealed that the USA isolate did not group with any of 8 characterized genera of *Chordopoxvirus*. Although, only a partial genome sequencing was performed (a limitation of the current study), the IsrRAPXV sequence was found to be related to the sequences of two recently identified poxviruses, the PTPV and SOPXV (Jacob et al., 2018).

Bats are reservoirs of numerous viral agents that are transmissible to humans and animals. With the exception of lyssaviruses, the majority of bat‐borne viruses reported to date do not appear to cause clinical disease in bats suggesting that the bat's tolerance to viral infection may be one of the key mechanisms by which they carry viruses that are pathogenic for humans (Pavlovich et al., 2018). It is interesting to note that our newly described virus, IsrRAPXV, was associated with typical poxvirus‐like lesions in a bat, namely, vesicular to nodular skin lesions. Egyptian fruit bats roost in large colonies that provide many opportunities for disease transmission including deposition of the virus on body surfaces followed by infection of abrasions or auto‐inoculation during grooming.

The zoonotic potential of IsrRAPXV is unknown. Although no pox infections have been reported in humans exposed to this bat, it would be prudent for professionals working with bats to wear protective clothes and gloves when handling them.

## References

[vms3233-bib-0001] Baker, K. S. , Leggett, R. M. , Bexfield, N. H. , Alston, M. , Daly, G. , Todd, S. , … Murcia, P. R. (2013). Metagenomic study of the viruses of African straw‐coloured fruit bats: Detection of a chiropteran poxvirus and isolation of a novel adenovirus. Virology, 441, 95–106. 10.1016/j.virol.2013.03.014 23562481 PMC3667569

[vms3233-bib-0002] Baker, K. S. , & Murcia, P. R. (2014). Poxviruses in Bats … so What? Viruses, 6, 1564–1577. 10.3390/v6041564 24704730 PMC4014710

[vms3233-bib-0003] Calisher, C. H. , Childs, J. E. , Field, H. E. , Holmes, K. V. , & Schountz, T. (2006). Bat: Important reservoir hosts of emerging viruses. Clinical Microbiological Review, 19, 531–545. 10.1128/CMR.00017-06 PMC1539106 16847084

[vms3233-bib-0004] Changula, K. , Kajihara, M. , Mori‐Kajihara, A. , Eto, Y. , Miyamoto, H. , Yoshida, R. , … Squarre, D. (2018). Seroprevalence of Filovirus Infection of Rousettus aegyptiacus Bats in Zambia. Journal of Infectious Diseases, 218 (suppl_5), S312–S317. 10.1093/infdis/jiy266 29889270

[vms3233-bib-0005] Ehelers, B. , Borchers, K. , Grund, C. , Frolich, K. , Ludwig, H. , & Buhk, H. J. (1999). Detection of new DNA polymerase gene of known and potentially novel herpesviruses by PCR with degenerate and deoxyinosine substituted primers. Virus Gene, 18, 211–220. 10.1023/a:1008064118057 10456789

[vms3233-bib-0006] Emerson, G. L. , Nordhausen, R. , Garner, M. M. , Huckabee, J. R. , Johnson, S. , Wohrle, R. D. , … Carroll, D. S. (2013). Novel poxvirus in big brown bats, northwestern United States. Emerging Infectious Disease, 19, 1002–1004. 10.3201/eid1906.121713 PMC3713833 23735421

[vms3233-bib-0007] Erster, O. , Melamed, S. , Paran, N. , Weiss, S. , Khinich, Y. , Gelman, B. , … Laskar‐Levy, O. (2018). First Diagnosed Case of Camelpox Virus in Israel. Viruses, 10(2), E78 10.3390/v10020078 29438294 PMC5850385

[vms3233-bib-0008] Jacob, J. M. , Subramaniam, K. , Tu, S.‐L. , Nielsen, O. , Tuomi, P. A. , Upton, C. , & Waltzek, T. B. (2018). Complete genome sequence of a novel sea otterpox virus. Virus Genes, 54, 756–767. 10.1007/s11262-018-1594-8 30225673

[vms3233-bib-0009] Levin, E. , Dolev, A. , & Solt, I. (2010). Bats in Israel: Is there a reason for medical concern? Harefuah, 149, 537–41, Review. 21341436

[vms3233-bib-0010] Li, Y. , Meyer, H. , Zhao, H. , & Damon, I. K. (2010). GC Content‐Based Pan‐Pox Universal PCR Assays for Poxvirus Detection. Journal of Clinical Microbiology, 48, 268–276. 10.1128/JCM.01697-09 19906902 PMC2812294

[vms3233-bib-0011] McLelland, D. J. , Reardon, T. , Bourne, S. , Dickason, C. , Kessell, A. , & Boardman, W. (2013). Outbreak of Skin Nodules Associated with *Riouxgolvania* *beveridgei* (Nematoda: Muspiceida) in the Southern Bentwing Bat (*Miniopterus* *schreibersii* *bassanii*), South Australia. Journal of Wildlife Diseases, 49, 1009–1013. 10.7589/2012-11-288 24502731

[vms3233-bib-0012] Menasherow, S. , Rubinstein‐Giuni, M. , Kovtunenko, A. , Eyngor, Y. , Fridgut, O. , Rotenberg, D. , … Stram, Y. (2014). Development of an assay to differentiate between virulent and vaccine strains of lumpy skin disease virus (LSDV). Journal of Virological Methods, 199, 95–101. 10.1016/j.jviromet.2013.12.013 24462845

[vms3233-bib-0013] O’Dea, M. A. , Tu, S. L. , Pang, S. , Ridder, T. D. , Jackson, B. , & Upton, C. (2016). Genome characterization of a novel poxvirus from a flying fox: Evidence for a new genus? Journal of General Virology, 97, 2363–2375. 10.1099/jgv.0.000538 27389615

[vms3233-bib-0014] Pavlovich, S. S. , Lovett, S. P. , Koroleva, G. , Guito, J. C. , Arnold, C. E. , Nagle, E. R. et al. (2018). The Egyptian rousette genome reveals unexpected features of Bat antiviral immunity. Cell, 173, 1–13. 10.1016/j.cell.2018.03 29706541 PMC7112298

[vms3233-bib-0015] Tuomi, P. A. , Murray, M. J. , Garner, M. M. , Goertz, C. E. C. , Nordhausen, R. W. , Burek‐ Huntington, K. A. et al. (2014). Novel poxvirus infection in northern and southern sea otters (*Enhydra* *lutriskenyoni* and *Enhydra* *lutris* *neiris*), Alaska and California USA. Journal of Wildlife Diseases, 50, 607–615. 10.7589/2013-08-217 24807180

